# Involvement of Membrane Progestin Receptor Beta (mPRβ/Paqr8) in Sex Pheromone Progestin-Induced Expression of Luteinizing Hormone in the Pituitary of Male Chinese Black Sleeper (*Bostrychus Sinensis*)

**DOI:** 10.3389/fendo.2018.00397

**Published:** 2018-07-18

**Authors:** Yu Ting Zhang, Wan Shu Hong, Dong Teng Liu, Heng Tong Qiu, Yong Zhu, Shi Xi Chen

**Affiliations:** ^1^State Key Laboratory of Marine Environmental Science, College of Ocean and Earth Sciences, Xiamen University, Fujian, China; ^2^Department of Biology, East Carolina University, Greenville, NC, United States; ^3^Fujian Collaborative Innovation Center for Exploitation and Utilization of Marine Biological Resources, Fujian, China; ^4^State-Province Joint Engineering Laboratory of Marine Bioproducts and Technology, Xiamen University, Fujian, China

**Keywords:** sex pheromone, progestin, luteinizing hormone, membrane progestin receptor, olfactory sensory neuron

## Abstract

Our previous studies showed that 17α, 20β-dihydroxy-4-pregnen-3-one (DHP) acted as a sex pheromone to induce reproductive success in Chinese black sleeper (*Bostrychus sinensis*), but its functional mechanism remains unclear. In the present study, we cloned the cDNAs of the gonadotropin subunits (*cg*α*, fsh*β, and *lh*β), and found that, in exposure to 5 nM DHP, transcript levels of *lh*β significantly increased in the pituitary at 6 h post exposure; plasma 11-KT levels increased at 24 h post exposure in mature male fish. In contrast, DHP exposure failed to increase the transcript levels of *lh*β in the pituitary of immature male fish, suggesting that the responsiveness to DHP depends on reproductive status. Interestingly, expression of progestin and adipoQ receptor 8 (*paqr8*, also known as mPRβ) and progesterone receptor membrane component 2 significantly increased in the olfactory rosette of male fish at late meiosis stage following a co-injection of human chorionic gonadotropin (HCG) and luteinizing hormone releasing hormone-A_3_ (LHRH-A_3_), while no increases of other progestin receptors were observed. Moreover, Paqr8 protein was localized in the dendritic knobs of the olfactory sensory neurons, which were activated following the *in vivo* exposure to DHP. The DHP-induced expression of *lh*β in pituitary was not inhibited by RU486, an antagonist of nuclear progesterone receptor. Taken together, our results suggested that sex pheromone DHP increased the expression of *lh*β transcript in the pituitary and plasma 11-KT levels of mature male, important for reproduction; and Paqr8 might be involved in responding to sex pheromone DHP in the olfactory rosette of male *B. sinensis*.

## Introduction

Many species of teleost uses two types of sex pheromones for achieving reproductive success: primer pheromones evoke changes in the endocrine or physiological state of conspecifics; and releaser pheromones induce rapid behavioral responses (1–4). 17α,20β-dihydroxy-4-pregnen-3-one (DHP), a maturation-inducing steroid in many teleost species, was first identified as a sex pheromone in goldfish *Carassius auratus* (1, 2, 5). Preovulatory female goldfish release DHP into the external environment by the gills and urine to induce a surge of luteinizing hormone (Lh) and promote milt production in the male fish (6). Since then, studies in different teleosts have added multiple lines of evidence for DHP as a sex pheromone (7–9). In goldfish, the molecular mechanisms and neural circuits underlying the pheromonal action of DHP have been well studied. Exposure to DHP activates a population of neurons in ventral pre-optic area (POA) that plays important roles in reproduction, and triggers immediate increases in gonadotropin-releasing hormone (GnRH) transcript in the telencephalon, plasma Lh and milt volume in male goldfish (10–12).

The teleost olfactory organ comprises an anterior nostril, olfactory chamber and a posterior nostril. The olfactory rosette with olfactory sensory neurons (OSNs) is located into the olfactory chamber, which connects with the exterior environment through the nostrils (13, 14). During olfactory stimulation, soluble odorants enter the olfactory chamber through the anterior nostril and flow over the olfactory rosette, where the molecules interact with olfactory receptors in OSNs. Until now, the olfactory receptor responding to sex pheromone DHP is not clear. Three types of progestin receptors have been identified so far, i.e., the nuclear progesterone receptor (PGR), progesterone receptor membrane component (PGRMC) and membrane progestin receptor (mPR). The mPRs belong to progestin and adipoQ receptor family (PAQR); consist of five subtypes: mPRα (PAQR7), mPRβ (PAQR8), mPRγ (PAQR5), mPRδ (PAQR6), and mPRε (PAQR9). Recently, *paqr5* and *paqr7* mRNAs were found in the olfactory epithelium of goldfish (15); and *paqr7* mRNA was found in the olfactory epithelium of Atlantic croaker *Micropogonias undulates* (16). In addition, Pgr immunoreactivity was observed in the olfactory epithelium in both sexes of the trout *Salmo trutta fario* (17). These results suggest that progestin receptors might be involved in responding to DHP in the olfactory rosette of fish.

The Chinese black sleeper (*Bostrychus sinensis*) belongs to the family Eleotridae, suborder Eleotroidei. This species is a burrowing fish and is of commercial importance in the southeastern regions of China (18). It is a seasonal breeding fish. Females and males live individually in their own burrows during the non-spawning season. During the spawning season, a pair of fish spawns together inside one burrow (18). Our previous studies showed that artificial nests with a DHP-releasing tube attracted more males and females and led to higher percentages of spawning than those of the control (18). Mature male *B. sinensis* displayed greater electro-olfactogram (EOG) response to DHP than immature fish (19). All known progestin receptors were expressed in the olfactory rosette, and the expression levels of the progestin receptors changed in the olfactory rosette of male fish during reproductive cycle (20). These results from our previous studies suggest that DHP is a sex pheromone in *B. sinensis*. However, the molecular mechanism underlying the pheromonal action of DHP on reproduction in *B. sinensis* is not clear.

In the present study, we first investigated the primer pheromonal effects of DHP on the expression of gonadotropin (GtH) subunits mRNAs in the pituitary and plasma 11-KT levels, both of which are important for testicular maturation. Thereafter, we examined the potential progestin receptor responding to DHP in the olfactory rosette of male *B. sinensis*.

## Materials and methods

### Experimental fish and chemicals

Male adult Chinese black sleeper *B. sinensis* were collected from Dadeng island, Fujian, China with the body length range of 150–169 mm and the body weight range of 72–108 g. Stock solutions of DHP and RU486 (Sigma–Aldrich, China) were prepared by dissolving the respective chemical compounds in 100% ethanol and stored at −20°C until use. All experimental protocols were reviewed and approved by the Institute Animal Care and Use Committee of Xiamen University.

### cDNA cloning of GtH subunits

Total RNA of pituitary was extracted using the RNAzol reagent (Molecular Research Center Inc. Cincinnati, OH, USA) and reverse transcribed into first strand cDNA using SMART RACE cDNA amplification kit (Clontech, Japan) following the manufacturer's instructions. Specific primers were designed according to previously reported GtH subunits sequences in teleost and the transcriptome data of *B. sinensis* brain. The PCR amplification was carried out in 20 μl volume under the following cycling conditions: 94°C for 3 min (1 cycle); 94°C for 30 s, 56°C for 30 s and 72°C for 1 min (35 cycles) followed by a final extension step at 72°C for 10 min. All PCR products were purified from agarose gel and sub-cloned into a pMD19-T vector (TAKARA, Japan), and then transformed into DH5α competent *E. coli* cells (Promega, Madison, WI, USA). Several positive clones were selected, their plasmid DNA were purified and prepared for DNA sequencing (Invitrogen Ltd, Guangzhou, China). Based on the partial cDNA sequence obtained, nested gene-specific primers were designed for 5′- and 3′-RACE (Supplemental Table 1). The first PCR amplification for 5′ or 3′ RACE was performed using a universal primer in the kit and a gene specific primer. If no specific band was obtained, these initial 5′ or 3′ RACE products were diluted and used for nested PCR amplifications with gene-specific nested primers, in combination with a nested universal primer. All RACE reactions were carried out following the manufacturer's instructions. RACE products were sub-cloned and sequenced as described above.

### Tissue specific expression of GtH mRNAs

Four adult male and female fish were anesthetized and humanely decapitated. The brains were trimmed to collect the olfactory bulb, telencephalon, diencephalon, mesencephalon, cerebellum, and medulla oblongata separately. The olfactory rosette, gill, heart, intestine, liver, spleen, skin, muscle, and gonad were also collected and immediately dipped into liquid nitrogen and stored at −80°C until analyses. Total RNA was extracted from the tissue samples using the RNAzol. Five hundred nanogram of total RNA from each tissue type was used for the synthesis of the first strand cDNAs using the RevertAid first strand cDNA synthesis kit (Thermo Scientific, USA). Real-time qPCR was performed as described in the section Real-Time qPCR.

### Determination of GtH subunits mRNAs during spermatogenesis

Male fish were anesthetized and humanely decapitated each month from December 2014 to July 2015. The pituitary and one testis were collected and immediately dipped into liquid nitrogen and stored at −80°C. Total RNA extraction and cDNA synthesis were conducted as described in the section cDNA Cloning of GtH Subunits. Real-time qPCR was performed as described in the section Real-Time qPCR. The other testis was fixed in Bouin's fixative overnight for conventional histology examination. The fixed testes were dehydrated through a graded series of ethanol concentrations (70–100%), embedded in paraplast (Leica, Germany), and 5 μm sections were obtained on a retracting microtome and stained with hematoxylin. Classification of male maturation state in *B. sinensis* was modified from the previous study (20). The testicular development was classified into five stages according to analyses of histology sections: stage I (spermatogonial proliferation stage), stage II (early meiosis stage), stage III (mid meiosis stage), stage IV (late meiosis stage), stage V (maturation stage) (Table 1).

**Table 1:** Classification of male maturation state in *B. sinensis* [modified from (20)].

**Stage**	**Classification**	**Microscopic appearance**
I	Spermatogonial proliferation stage	Spermatogonia are observed in testis
II	Early meiosis stage	Spermatocytes are observed in testis
III	Mid meiosis stage	Spermatids are observed in testis
IV	Late meiosis stage	All types of germ cells are observed
V	Maturation stage	Numbers of spermatogonia and spermatocytes are declining, and the lobule lumen is filled with spermatozoa

### *In vivo* exposure to DHP and RU486

Immature (at stage III) and mature (at stage V) male fish were used in DHP exposure experiment. Plastic tanks (15 L) were used as the experimental containers. Fish were group housed in tanks with 3 individuals in each tank for at least 24 h before the experiment. Salinity was maintained at 15 ‰. The fish were not fed during the experimental period. The vehicle or DHP were added into the tank water to reach final concentrations of DHP of 0, 0.5, or 5 nM. The fish were then deeply anesthetized and humanely sacrificed at 3, 6, 12, and 24 h following DHP exposure. Pituitary was collected for determination of GtH subunits mRNAs. Blood samples were collected for 11-KT measurement at 12 and 24 h after exposure to DHP. To study whether the effects of DHP on *lh*β transcript expression is via Pgr signal pathway, mature male fish were exposed to DHP (5 nM) with Pgr antagonist RU486 (100 nM and 1 μM) for 6 h. A control group was exposed only to 1 μM RU486 for 6 h. At the end of exposure, the fish were anesthetized and their pituitary samples were collected. To examine which OSNs were activated by DHP, the fish were exposed to 5 nM DHP for 10 min. Then the olfactory rosette was removed and fixed in 4% paraformaldehyde (PFA). Total RNA extraction and cDNA synthesis for the pituitary were conducted as described in the section cDNA Cloning of GtH Subunits and the real-time qPCR was performed as described in the section Real-Time qPCR. The plasma 11-KT levels were measured as described in the section Measurement of Plasma 11-KT. The immunohistochemistry was performed as described in the section Antibody Production, Western Blot, and Immunohistochemistry.

### Transactivation assays for *B. sinensis* Pgr

HEK293T cells were cultured in 6-cm dishes in Dulbecco modified Eagle medium (DMEM) supplemented with 10% v/v fetal bovine serum (FBS), and penicillin/streptomycin (Gibco) at 37°C in a 5% CO_2_ incubator. For the transient transfection, cells were co-transfected with 120 ng of pRL-TK vector containing the Renilla luciferase reporter gene (as a control for transfection efficiency), 400 ng of the *B. sinensis* Pgr expression plasmid, and 1.2 μg of pGL3-MMTV-Luc plasmid, containing the mouse mammary tumor virus-long terminal repeat (MMTV-LTR) and Firefly luciferase reporter gene (21) using Lipofectamine 3000 (Life Technologies, USA). Transfected cells were lifted and reseeded in 24-well plates (NEST, USA) 8 h after the transfection. After 12 h, cells were washed, and incubation medium was replaced with a transactivation assay medium (DMEM without phenol red, supplemented with 10% v/v charcoal-stripped FBS, and penicillin/streptomycin) containing various concentrations of DHP (100 pM−10 μM) and PGR antagonist RU486 (1–100 μM). Cells were harvested to determine Firefly and Renilla luciferase activities after another 24 h incubation. Firefly and Renilla luciferase activities were measured using the Dual Luciferase Assay System (Promega, USA) on a luminometer (Promega, USA). Firefly luciferase data were normalized to Renilla luciferase data. After normalization for transfection efficiency, induction factors were calculated as the ratios of the average value of the luciferase value of the steroids stimulated samples vs. vehicle treated samples.

### Co-injection of HCG and LHRH-A_3_

Male fish (152–160 mm body length and 93–100 g body weight) at stage IV were randomly divided into two groups with 8 individuals each. Fish were then acclimated to environment for 1 day before the experiment. In the experimental group each fish was injected intraperitoneally with an aliquot of 200 μl PBS containing 700 IU human chorionic gonadotropin (HCG) and 7 μg luteinizing hormone releasing hormone-A_3_ (LHRH-A_3_) (Ningbo Shansheng Pharmaceutical Co., China), an efficient technical to induce maturation in male *B. sinensis* (22). A second injection with same doses was administrated at 24 h after the first injection. The olfactory rosettes were surgically dissected and immediately frozen in liquid nitrogen at 24 h following second injection. Frozen samples were stored at −80°C until gene expression analyses.

### Antibody production, western blot, and immunohistochemistry

Specific mouse polyclonal antibody was generated against synthetic 15-oligo peptide (MPGDILQRLTTLTL) derived from the N-terminal of Paqr8 of *B. sinensis*. The peptide was linked to keyhole limpet hemocyanin (KLH). After five intradermal injections (200 μg per injection in Freund's adjuvant), antibody specify was verified by detection of Paqr8 protein in transiently transfected HEK293T and in *B. sinensis* olfactory rosette and testes samples by Western blot analysis.

Western blot assay was performed as previous described (23). In brief, total protein samples were extracted by immediately placing freshly excised tissues or cells into 2 × SDS buffer, denatured by boiling for 5 min, and then cooled on ice. Protein samples were loaded and separated using a 12% SDS-PAGE gel and were transferred onto a PVDF membrane. The membrane was blocked in TBST containing 0.1% (v/v) tween-20 and 5% (w/v) defatted milk powder for 1 h at room temperature (RT), before the membrane was incubated with Paqr8 antibody (1:1000, v/v) for 12 h at 4°C. After five washes with TBST, the membrane was incubated for 1 h at RT with the horseradish peroxidase (HRP)-conjugated secondary antibody (1:1000, v/v). A chemiluminescence detection kit (TransGen, China) was used to detect signals on the PVDF membrane.

For immunohistochemistry, the deparaffined sections of olfactory rosette were first incubated with normal mouse serum (1:1000) as control, primary antiserum against Paqr8 (1:1000) or pERK (1:400, Cell Signaling Technology, Danvers, USA) overnight (~18 h) at 4°C, then the horseradish peroxidase was introduced using the ABC method (Vector Laboratories Inc., Burlingame, CA, USA). Thereafter, a TSA Fluorescein/Cyanine 5 system was used for single or double color detection of target proteins according to the protocol (PerkinElmer, USA) (23). Sections were then washed in PBS and mounted with VectaShield containing DAPI (Vector Laboratories).

### Real-time qPCR

Specific primers for quantitative PCR (qPCR) analyses of target genes (*cg*α, *fsh*β, and *lh*β) were designed and examined for their specificity and amplification efficiency on serial dilutions of respective target gene plasmid DNA (10^3^-10^8^ copies/μl) (Supplemental Table 1). All qPCR was performed in a 20 μl reaction mixture on the 7500 FAST real-time PCR detection system (Applied Biosystems, USA) using default settings. The relative mRNA levels of the target genes were determined using the comparative Ct method (24) with the β*-actin* gene as an internal control. Transcript levels of β*-actin* gene were stable (Supplemental Figure 1). The specificity and efficiency of the specific primers for β*-actin* and progestin receptors have been described and validated in previous studies (25, 20).

### Measurement of plasma 11-KT

Plasma were obtained by centrifugation of fresh harvested blood at 1000 *g* for 15 min at 4°C, and stored at −80°C until analysis. Plasma 11-KT levels were determined using a protocol described previously (26) and an EIA kit, purchased from the Caymen Chemical Company (Ann Arbor, Michigan, USA).

### Statistical analysis

Data were presented as means ± standard error of the mean (SEM). Depending on the experimental setup, data were analyzed using either Student' s *t*-test or one-way ANOVA followed by Fisher' s PLSD post hoc test to assess statistical differences among the individual groups using the SPSS (version 21.0) statistical software package.

## Results

### Expression patterns of GtH subunits transcripts in the pituitary during spermatogenesis

GtH subunits, *cg*α (GenBank accession number: MG866074), *fsh*β (GenBank accession number: MG866075), and *lh*β (GenBank accession number: MG866076) were cloned from the pituitary of *B. sinensis* (Supplemental Figure 2) and phylogenetic analyses of *B. sinensis* Cgα, Fshβ, and Lhβ clearly grouped them together with those GtH subunits from teleosts (Supplemental Figure 3). The expression of *cg*α, *fsh*β, and *lh*β transcripts were mainly in the pituitary (Supplemental Figure 4).

**Figure 1:**
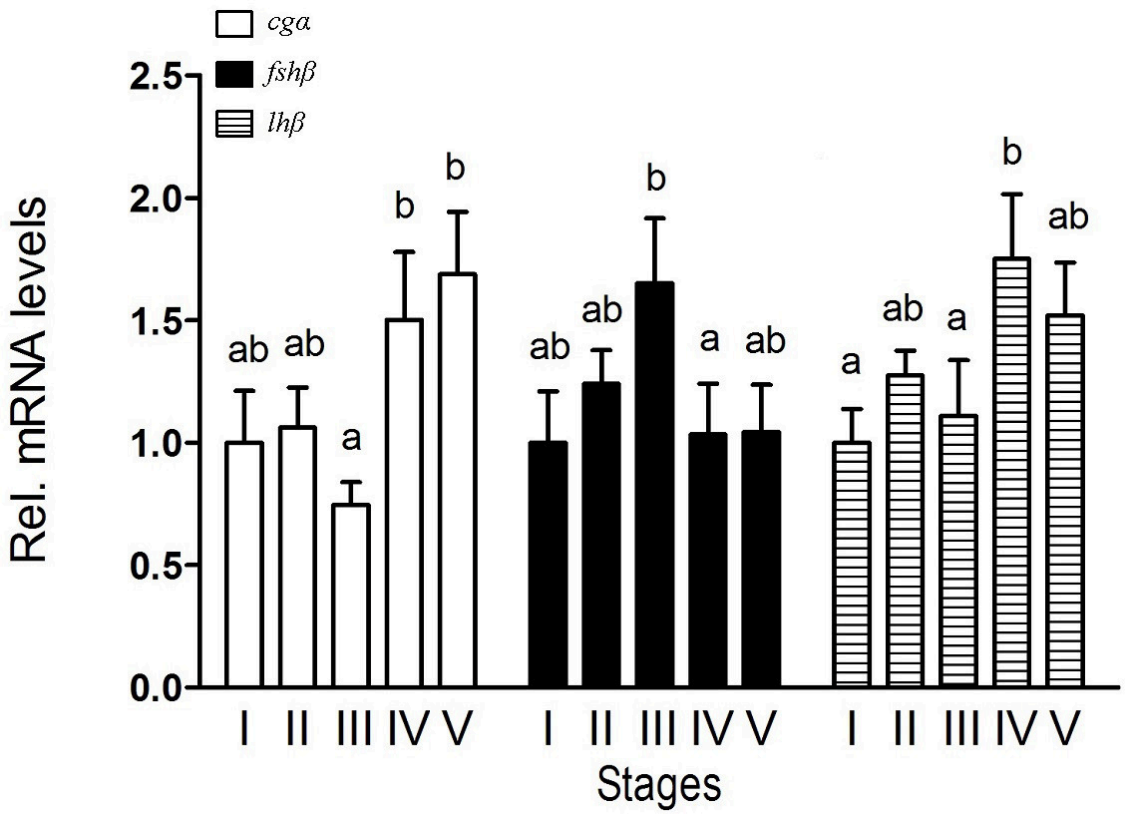
Expression of *cg*α, *fsh*β, and *lh*β in the pituitary of male *B. sinensis* during spermatogenesis. Stage I (Spermatogonial proliferation stage), Stage II (Early meiosis stage), Stage III (Mid meiosis stage), Stage IV (Late meiosis stage), and Stage V (Maturation stage). Data are expressed as the mean ± SEM (*n* = 6). Bars marked with different letters are significantly different from each other (*p* < 0.05).

During spermatogenesis, different expression patterns of *fsh*β and *lh*β transcripts in the pituitary were observed. Transcript levels of *fsh*β peaked at stage III and thereafter decreased, while that of *lh*β peaked at stage IV. For *cg*α transcript, the highest levels in the pituitary were found at stage IV and V (Figure 1). In addition, similar expression patterns of GtH transcripts were observed in the testis (Supplemental Figure 5).

### DHP exposure increased the expression of pituitary *lhβ* and plasma 11-KT

**Figure 2:**
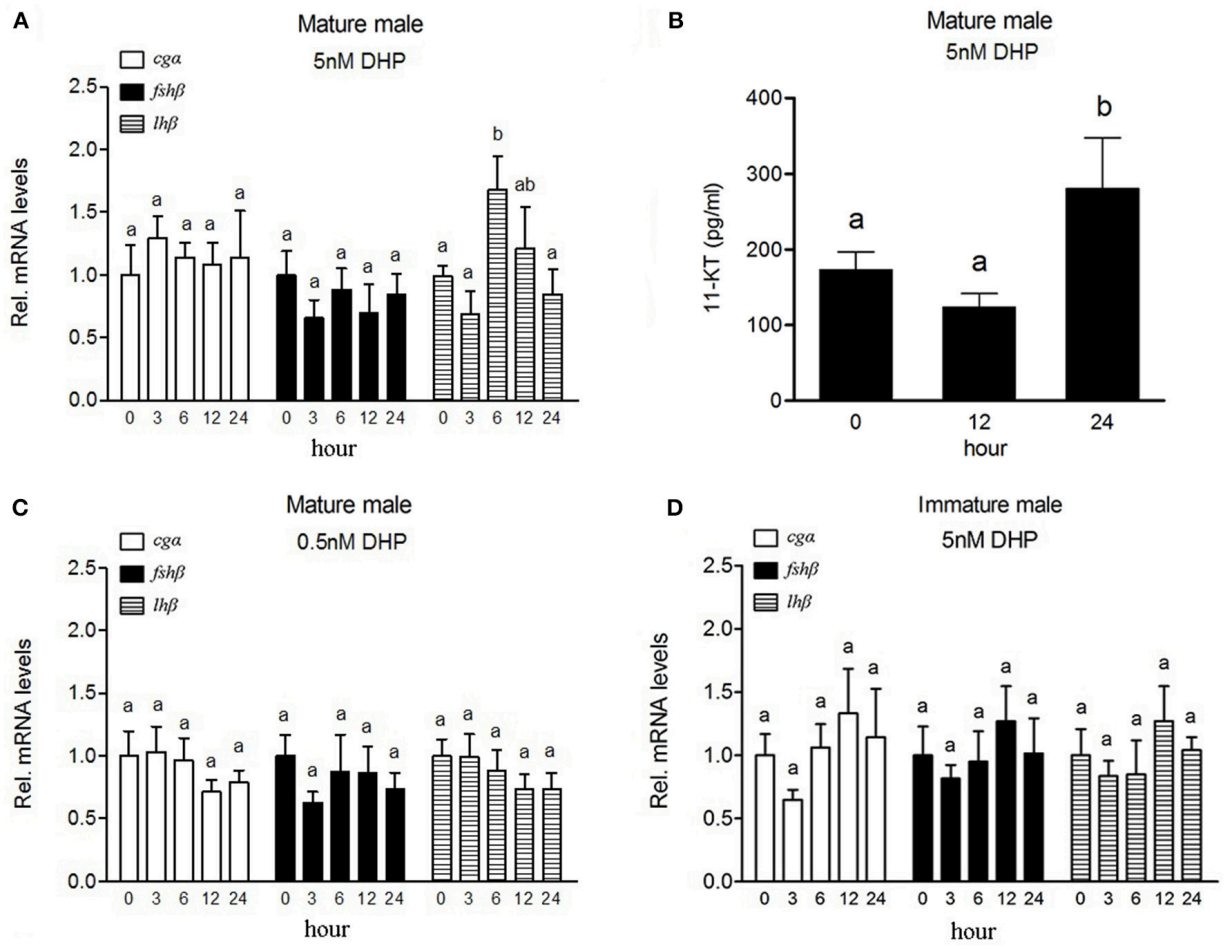
Effects of DHP exposure on the expression of GtH subunits mRNAs in the pituitary and plasma 11-KT levels in male *B. sinensis*. Changes of *cg*α, *fsh*β, and *lh*β mRNA levels in the pituitary after 5 nM **(A)** and 0.5 nM **(C)** DHP exposure, and changes of plasma 11-KT levels after 5 nM DHP exposure **(B)** in mature male fish (at stage V). Changes of *cg*α, *fsh*β, and *lh*β mRNA levels in the pituitary of immature fish (at stage III) after 5 nM DHP exposure **(D)**. All data are expressed as the mean ± SEM (*n* = 6). Bars marked with different letters are significantly different from each other (*p* < 0.05).

The expression of *lh*β transcript increased significantly in the pituitary of mature male fish (at stage V) exposed to 5 nM DHP for 6 h (Figure 2A), while expression of *cg*α or *fsh*β had no significant change in the pituitary under same conditions. A significant increase of plasma 11-KT levels was observed in mature fish exposed to 5 nM DHP for 24 h (Figure 2B). Exposure to a low dose of DHP (0.5 nM) did not induce any significant change of any GtH subunit in the pituitary (Figure 2C). In addition, DHP exposure (5 nM) failed to increase *lh*β transcript levels in the pituitary of immature fish (at stage III) (Figure 2D).

### Expression of progestin receptors transcripts in the olfactory rosette of male fish at late meiosis stage in response to co-injection of HCG and LHRH-A_3_

**Figure 3:**
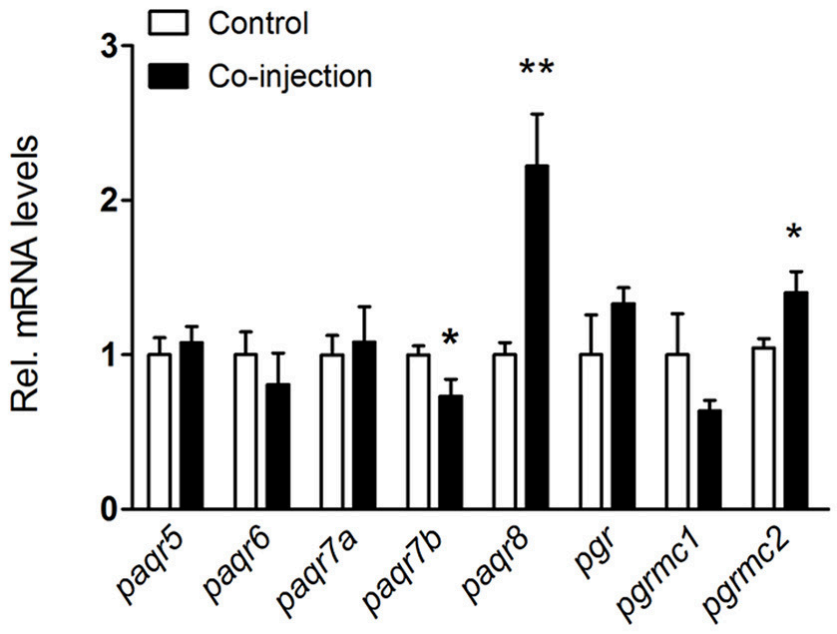
Expression changes of nine progestin receptors in the olfactory rosette of nearly mature male *B. sinensis* (at stage IV) after co-injection of HCG and LHRH-A_3_. All data are expressed as the mean ± SEM (*n* = 8). *p < 0.05; **p < 0.01.

In our previous study, the expression of some progestin receptors in the olfactory rosette increased and stayed at high levels when testis enters maturation stage (20). In the present study, we examined the expression levels of all progestin receptors in male fish after HCG and LHRH-A_3_ treatment which promotes testicular maturation (from stage IV to V). After co-injection of HCG and LHRH-A_3_, the expression of *paqr8* and *pgrmc2* transcripts significantly increased (~2.5-fold for *paqr8* and ~1.5-fold for *pgrmc2*), while a decrease of *paqr7b* and no changes of other progestin receptors were observed in the olfactory rosette (Figure 3).

### Cellular localization of pERK and Paqr8 in the olfactory rosette

**Figure 4:**
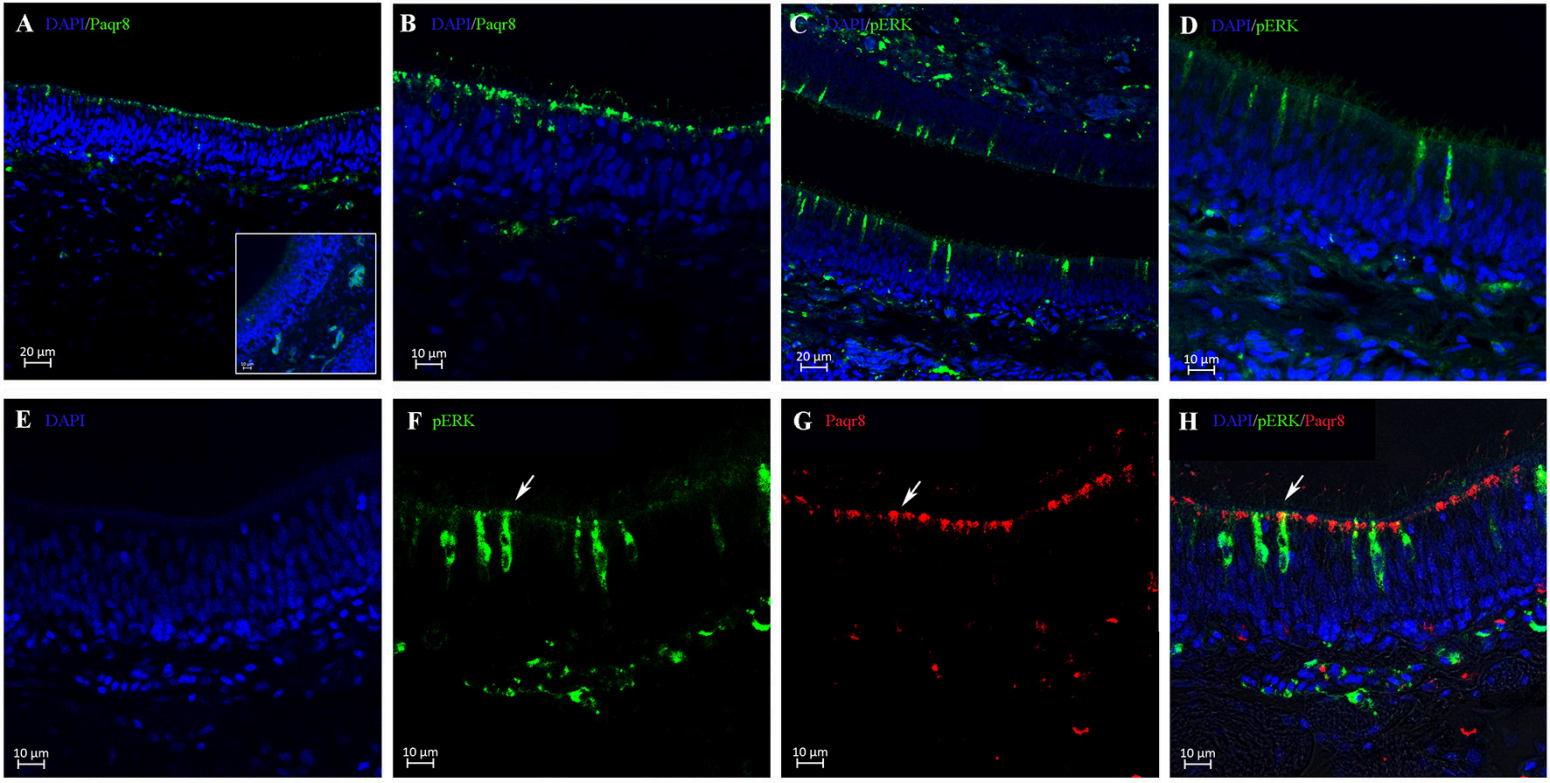
Cellular localization analysis of Paqr8 and pERK in the olfactory rosette of mature *B. sinensis* (at stage V) using confocal microscope imaging. **(A,B)** Paqr8 immunostaining of the olfactory rosette sections. The inset in panel A shows negative control; note the absence of specific staining in the dendritic knobs in the apical surface. **(C,D)** pERK immunostaining of the olfactory rosette sections exposed to 5 nM DHP. **(E–H)** pERK (green) and Paqr8 (red) double-colored fluorescent immunostaining of the olfactory rosette exposed to 5 nM DHP for 10 min. White arrows indicate co-localization of pERK and Paqr8 signals.

A single and specific band with the expected size (41 kDa) was observed in olfactory rosette and testis tissues using polyclonal antibody generated against *B. sinensis* Paqr8 protein (Supplemental Figure 6). Results of immunohistochemistry showed that Paqr8 protein was expressed in the olfactory epithelium in male *B. sinensis*, and was widely distributed in the dendritic knobs in the apical surface (Figures 4A,B). Following an *in vivo* exposure to 5 nM DHP for 10 min, the signals of phosphorylated ERK (pERK), a marker for neuronal activation, was observed in some OSNs, (Figures 4C,D), while few pERK signals were observed in vehicle-exposed control fish (Supplemental Figure 7). Importantly, double labeling of pERK and Paqr8 signals were observed in the same OSNs (Figures 4E–H).

### RU486 blocked Pgr mediated transcription in HEK293T cells but not the expression of *lhβ in vivo*

**Figure 5:**
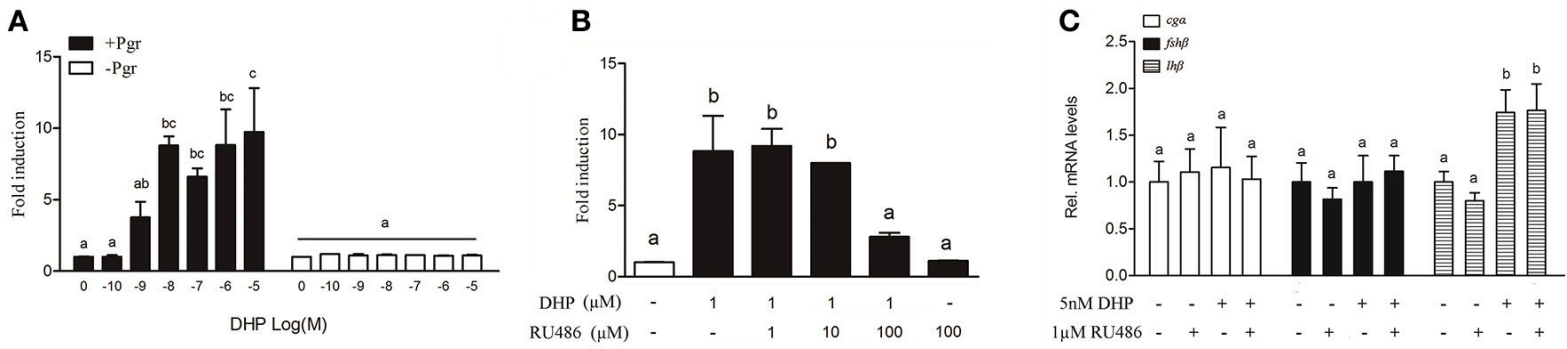
**(A)** DHP-induced transactivation properties of the *B. sinensis* Pgr in HEK293T cells. The cells were incubated for 24 h with increasing concentrations of DHP (100 pM to 1 μM). **(B)** Antagonistic effects of RU486 on Pgr-mediated transactivation. The cells were incubated for 24 h with DHP at 1 μM with or without 1, 10, or 100 μM RU486. **(C)** Effects of RU486 on DHP-stimulated pituitary GtH expression in mature male *B. sinensis* (at stage V). Data are expressed as the mean ± SEM (*n* = 6). Bars marked with different letters are significantly different from each other (*p* < 0.05).

A dose-dependent, Pgr-mediated activation of the MMTV promoter was observed in HEK293T cells transfected with *B. sinensis pgr* (Figure 5A). Luciferase activity induced by DHP (1 μM) was inhibited by RU486 (100 μM) in HEK293T cells (Figure 5B). Therefore, RU486 was used to determine if DHP-induced *lh*β expression was mediated by Pgr in the olfactory rosette. The results showed that neither 100 nM (data not shown) nor 1 μM RU486 (Figure 5C) inhibited the *in vivo* stimulatory effects of DHP (5 nM) on the expression of *lh*β in mature male *B. sinensis*.

## Discussion

As a primer sex pheromone released from female fish, one possible physiological function of DHP is to stimulate GtHs expression levels of mature males (27). In the present study, after cloning the cDNAs of GtH subunits, we observed that the transcript levels of *fsh*β and *lh*β showed different expression patterns in the pituitary of *B. sinensis* during spermatogenesis. Transcript level of *fsh*β was high at stage III and decreased afterwards, while transcript level of *lh*β started to increase at stage IV, which suggests Fsh may be mainly involved in early spermatogenesis, while Lh may be important for the final testicular maturation in male *B. sinensis*. Exposure of mature male *B. sinensis* to DHP for 6 h resulted in a significant increase of *lh*β but not *fsh*β in the pituitary. In zebrafish, DHP exposure (100 nM) stimulates the expression of both *fsh*β and *lh*β transcripts in the pituitary through an endocrine pathway (23). These stimulatory effects require high concentration of DHP (100 nM) and long exposure period (24 h) (23). In contrast, sex pheromones usually induce a rapid response at low concentrations (1, 2, 10, 28, 29). Considering the low concentration of DHP (5 nM), the rapid response to DHP (6 h) and DHP-activated OSNs were observed in the present study, it is most likely that the stimulatory effect of DHP on *lh*β is mainly mediated via the olfactory system in male *B. sinensis*. In addition, a significant increase of plasma 11-KT was observed 24 h after DHP exposure. In male teleost, Lh surge during spawning season induces an increase in the production of the testicular steroids, such as 11-KT and DHP. Both Lh and these steroids are involved in final maturation of male gametes and spermiation process in fish (30–33). Therefore, the increase of plasma 11-KT levels may reflect the plasma Lh rise due to the increase of pituitary *lh*β transcript levels in male *B. sinensis*. Taken together, the results from the present study suggest that DHP plays as a primer sex pheromone to stimulate pituitary *lh*β expression resulting in plasma Lh rise, followed by 11-KT rise; both Lh and 11-KT contribute to the reproductive success in *B. sinensis*.

The functional mechanism of DHP as sex pheromone in reproduction is well documented in teleost, but the molecular mechanisms for DHP detection in the olfactory rosette is not well understood. It is well known that signal transduction in the olfactory system begins with the binding of an odorant ligand to a receptor on the olfactory neuron cell surface, initiating a cascade of enzymatic reactions that results in the production of a second messenger and the eventual depolarization of the cell membrane (34). In goldfish, specific binding for DHP has been found in olfactory epithelium membrane preparations using radio-receptor assays (35). In the present study, we observed that DHP activated OSNs using the neuronal activation marker pERK, which suggests DHP interacts with olfactory receptors expressed in the OSNs. Pgr is suggested to be a candidate receptor for the exogenous DHP detection in teleost (15, 17). Besides its role in genome-mediated physiological processes, Pgr has also been implicated to mediate rapid and non-genomic progestin signaling (36). However, our previous study showed the expression of *pgr* transcript was much lower than those of other progestin receptors and dramatically declined in the olfactory rosette of male *B. sinensis* during testicular maturation (20). In the present study, we found that the DHP-induced increase of *lh*β in the pituitary was not inhibited by RU486. These results suggest that other types of progestin receptors in OSNs may be involved in the DHP-induced increase of *lh*β.

Our previous studies showed that the higher olfactory sensitivity to DHP was observed in mature fish than in immature fish (19) and the expression levels of some progestin receptors in the olfactory rosette increased and stayed at high levels when testis entered maturation stage (20). We suppose that the expression levels of progestin receptors responding to DHP should be positively regulated by hormones that promote testicular maturation. Therefore, in the present study, in order to provide a clue for the identification of progestin receptors involved in mediating the pheromonal effects of DHP, we examined the expression responses of progestin receptors to HCG and LHRH-A_3_ treatment, which could promote testicular maturation (from stage IV to V). The results showed that, among those progestin receptors showing increasing patterns when testis entered maturation stage, only *paqr8* and *pgrmc2* were stimulated by HCG and LHRH-A_3_. Interestingly, our previous study showed that the expression pattern of *paqr8* in the male olfactory rosette mirrored the changes of plasma DHP levels in females during the reproductive cycle. Furthermore, in the present study, we observed that Paqr8 protein was localized in the dendritic knobs of OSNs, which is similar to the cellular localization of Paqr8 protein in mouse olfactory epithelium (37). Some of the Paqr8 positive OSNs showed positive pERK signaling after DHP exposure, which indicates that the OSNs expressing Paqr8 are activated by DHP. Taken together, it is most likely that Paqr8 in the olfactory rosette is involved in responding to exogenous DHP.

DHP exposure failed to increase pituitary *lh*β transcript in immature male *B. sinensis*, which indicates that the responsiveness to DHP depends on reproductive status. This result agrees with previous study in goldfish (38), and our previous studies also showed that mature male *B. sinensis* displayed greater EOG response to DHP than immature male fish (19). It is suggested that the sensitivity of olfactory rosette to DHP is associated with the expression levels of DHP receptors. Study in mouse has shown that gonadal hormones may affect the response of vomeronasal organ neurons to chemosignals by altering levels of the receptors to which they bind (39). As the expression of *paqr8* in the olfactory rosette increased with the spontaneous (20) and artificial induction of (this study) testicular maturation, the changes of Paqr8 expression levels might account for different sensitivity to DHP between immature and mature male *B. sinensis*. Investigating the physiological mechanism for the increase of *paqr8* levels in mature male fish will be an important subject in further studies.

A decrease of *paqr7b* in the olfactory rosette was observed after the co-injection of HCG and LHRH-A_3_. Our previous study showed that the expression pattern of *paqr7b* in the male olfactory rosette slightly decreased at mature status, suggesting that the function of Paqr7b in the olfactory rosette might not be closely related to testicular maturation and spawning activity (20). In the present study, the co-injection of HCG and LHRH-A_3_, which enhances the mature status, inhibited the expression of olfactory *paqr7b*. This finding further suggests that the function of Paqr7b in the olfactory rosette might be irrelevant to testicular maturation and spawning activity. Furthermore, Paqr7b might disturb the function of Paqr8, because the native ligand of both two receptors is DHP. However, the mechanism of inhibitory effects of HCG and LHRH-A_3_ on the *paqr7b* expression is unclear so far. It will be an interesting issue to address in the future.

In summary, the present study provided clear evidence that DHP acted as a primer sex pheromone to stimulate pituitary *lh*β expression which resulted in an increase of plasma 11-KT levels via the olfactory system in mature male *B. sinensis*. The Paqr8 expressed in OSNs of olfactory epithelium might be involved in responding to DHP, and the responsiveness to DHP might depend on the expression levels of the Paqr8 in male *B. sinensis*.

## Author contributions

YTZ was involved in entire study. DTL analyzed results. HTQ performed antibody screen. YZ analyzed results and prepared manuscript drafting. SXC and WSH conceived and supervised the project, analyzed results, and prepared the manuscript.

### Conflict of interest statement

The authors declare that the research was conducted in the absence of any commercial or financial relationships that could be construed as a potential conflict of interest.
