# Supplementary material for: Involvement of Membrane Progestin Receptor Beta (mPRβ/Paqr8) in Sex Pheromone Progestin-Induced Expression of Luteinizing Hormone in the Pituitary of Male Chinese Black Sleeper (Bostrychus Sinensis)
Source: Front Endocrinol (Lausanne). 2018 Jul 18;9:397. doi: 10.3389/fendo.2018.00397 (PMC6058016; doi:10.3389/fendo.2018.00397)
Supplement: Supplementary file 1 [file Data_Sheet_1.PDF]

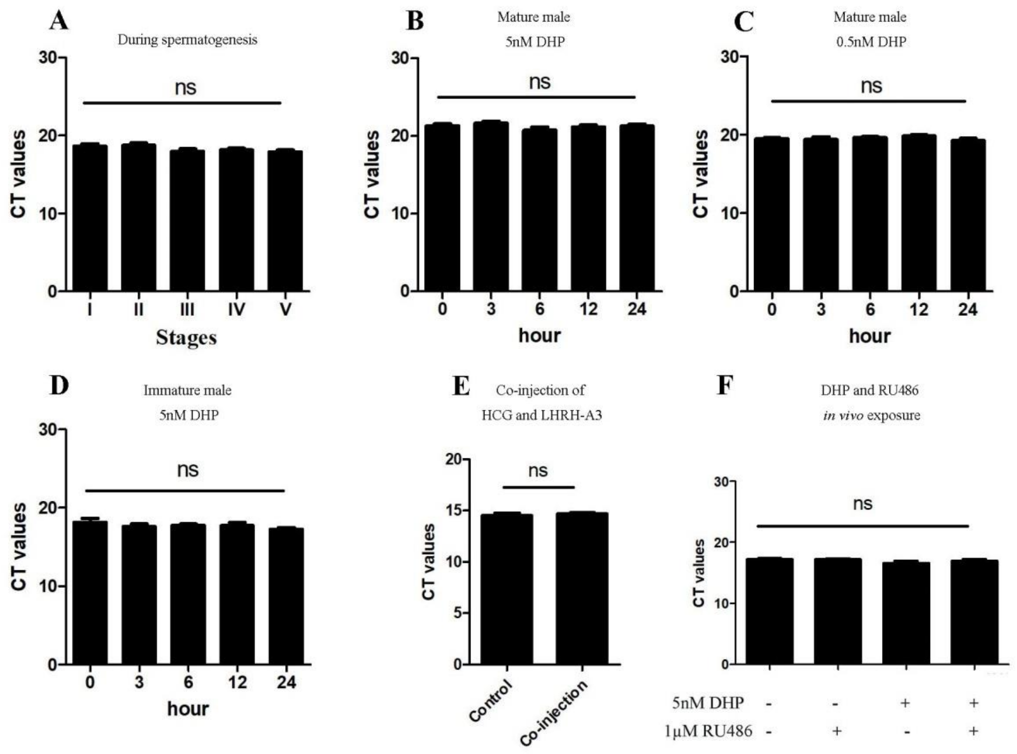

Supplemental Fig.1 CT values of house keeping gene *β-actin* in the present study. CT values of *β-actin* in the pituitary of male *B. sinensis* during spermatogenesis (A). CT values of *β-actin* in the pituitary of mature male fish (at stage V) after 5 nM (B) and 0.5 nM (C) DHP exposure, and that of immature male fish (at stage III) after 5 nM DHP exposure (D). CT values of *β-actin* in the olfactory rosette of male fish at stage IV after co-injection of HCG and LHRH-A_3 (E). CT values of *β-actin* in the pituitary of mature male fish (at stage V) after DHP and/or RU486 *in vivo* exposure (F). ns, not significant.

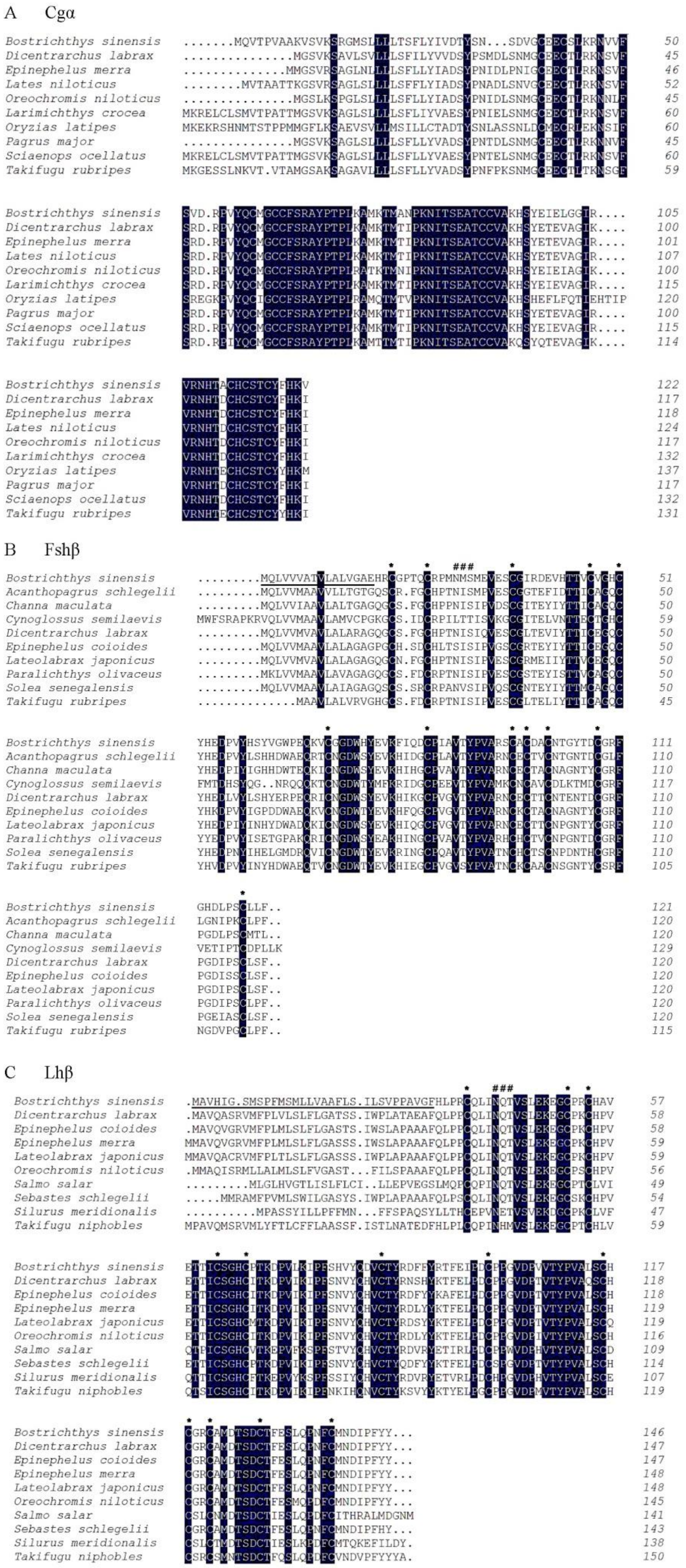

A Cgα
Bostrichthys sinensis
Dicentrarchus labrax
Epinephelus merra
Lates niloticus
Oreochromis niloticus
Larimichthys crocea
Oryzias latipes
Pagrus major
Sciaenops ocellatus
Takifugu rubripes
B Fshβ
Bostrichthys sinensis
Acanthopagrus schlegelii
Channa maculata
Cynoglossus semilaevis
Dicentrarchus labrax
Epinephelus coioides
Lateolabrax japonicus
Paralichthys olivaceus
Solea senegalensis
Takifugu rubripes
C Lhβ
Bostrichthys sinensis
Dicentrarchus labrax
Epinephelus coioides
Epinephelus merra
Lateolabrax japonicus
Oreochromis niloticus
Salmo salar
Sebastes schlegelii
Silurus meridionalis
Takifugu niphobles

Supplemental Fig.2 Alignment of *B. sinensis* Cgα (A), Fshβ (B) and Lhβ (C) deduced amino acid sequence. Predicted signal peptides are underlined. The conserved potential N-glycosylation site is marked with octothorpe. The conserved cysteine residues are marked with asterisks.

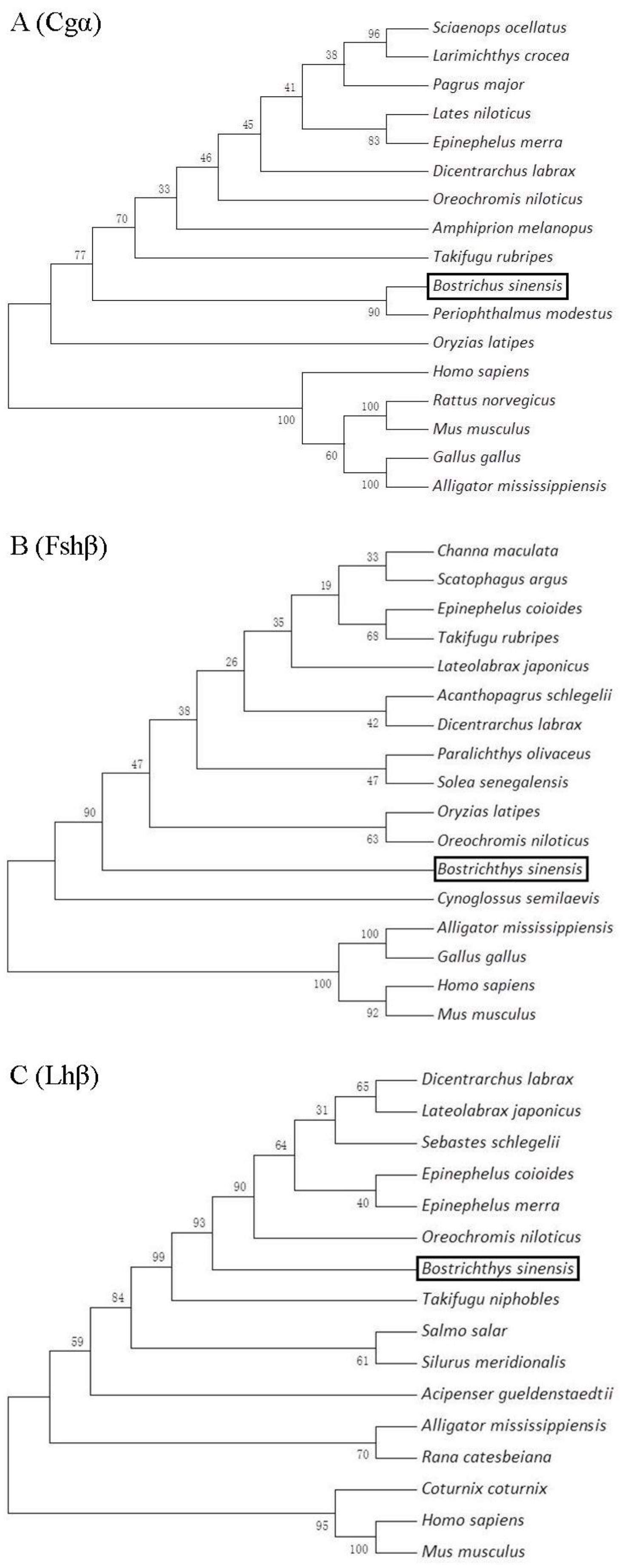
A (Cgα)
Sciaenops ocellatus
Larimichthys crocea
Pagrus major
Lates niloticus
Epinephelus merra
Dicentrarchus labrax
Oreochromis niloticus
Amphiprion melanopus
Takifugu rubripes
Bostrichus sinensis
Periophthalmus modestus
Oryzias latipes
Homo sapiens
Rattus norvegicus
Mus musculus
Gallus gallus
Alligator mississippiensis
B (Fshβ)
Channa maculata
Scatophagus argus
Epinephelus coioides
Takifugu rubripes
Lateolabrax japonicus
Acanthopagrus schlegelii
Dicentrarchus labrax
Paralichthys olivaceus
Solea senegalensis
Oryzias latipes
Oreochromis niloticus
Bostrichthys sinensis
Cynoglossus semilaevis
Alligator mississippiensis
Gallus gallus
Homo sapiens
Mus musculus
C (Lhβ)
Dicentrarchus labrax
Lateolabrax japonicus
Sebastes schlegelii
Epinephelus coioides
Epinephelus merra
Oreochromis niloticus
Bostrichthys sinensis
Takifugu niphobles
Salmo salar
Silurus meridionalis
Acipenser gueldenstaedtii
Alligator mississippiensis
Rana catesbeiana
Coturnix coturnix
Homo sapiens
Mus musculus

Supplemental Fig.3 Unrooted phylogenetic analysis of *B. sinensis* Cgα (A), Fshβ (B) and Lhβ (C). Multiple species' amino acid sequences of Cgα, Fshβ and Lhβ were aligned using Clustal W. GenBank accession numbers for sequence data analyzed are: *Sciaenops ocellatus* Cgα, ACZ37431.1; *Larimichthys crocea* Cgα, XP_010733202.1; *Pagrus major* Cgα, BAB18562.1; *Lates niloticus* Cgα, AKE14360.1; *Epinephelus merra* Cgα, BAJ05295.1; *Dicentrarchus labrax* Cgα, AAK49431.1; *Oreochromis niloticus* Cgα, NP_001266680.1; *Takifugu rubripes* Cgα, XP_003962541.1; *Amphiprion melanopus* Cgα, ACH43020.1; *Periophthalmus modestus* Cgα, BAF43299.1; *Oryzias latipes* Cgα, BAK61760.1; *Homo sapiens* Cgα, AAD13690.1; *Gallus gallus* Cgα, NP_001264950.1; *Rattus norvegicus* Cgα, NP_446370.2; *Mus musculus* Cgα, EDL05461.1; *Channa maculata* Fshβ, AAS01610.1；*Scatophagus argus* Fshβ, AQS95492.1; *Epinephelus coioides* Fshβ, AAO31971.1; *Takifugu rubripes* Fshβ, DAA06176.1; *Lateolabrax japonicus* Fshβ, AFN02626.1 ； *Acanthopagrus schlegelii* Fshβ, ADX31689.1 ； *Dicentrarchus labrax* Fshβ, AAN40506.1； *Paralichthys olivaceus* Fshβ, AAK58601.1； *Solea senegalensis* Fshβ, ABW81403.1； *Oryzias latipes* Fshβ, ABQ08583.1; *Oreochromis niloticus* Fshβ, NP_001266672.1; *Cynoglossus semilaevis* Fshβ, AFF59206.1 ； *Alligator mississippiensis* Fshβ, BAJ14506.1；*Gallus gallus* Fshβ, ACW82409.1；*Homo sapiens* Fshβ, ABQ57402.1; *Mus musculus* Fshβ, NP_032071.1; *Dicentrarchus labrax* Fshβ, AAN40507.1; *Lateolabrax japonicus* Fshβ, AFN02625.1; *Sebastes schlegelii* Fshβ, AAU14142.1; *Epinephelus coioides* Fshβ, AAN18041.1; *Epinephelus merra* Fshβ, BAJ05297.1; *Oreochromis niloticus* Fshβ, XP_003438397.1; *Takifugu niphobles* Fshβ, BAJ12080.1; *Salmo salar* Fshβ, NP_001167142.1; *Silurus meridionalis* Fshβ, AAY42269.2; *Acipenser gueldenstaedtii* Fshβ, AAP97490.1; *Alligator mississippiensis* Fshβ, BAJ14507.1; *Rana catesbeiana* Fshβ, AAY21812.1; *Coturnix coturnix* Fshβ, AAB30867.1; *Homo sapiens* Fshβ, AAL69719.1; *Mus musculus* Fshβ, EDL22853.1. The alignment was performed using MEGA 6.0 program and the Clustal W method. Then a phylogenetic tree was constructed using the neighbor-joining method with a bootstrap value of 1000 trials for each position.

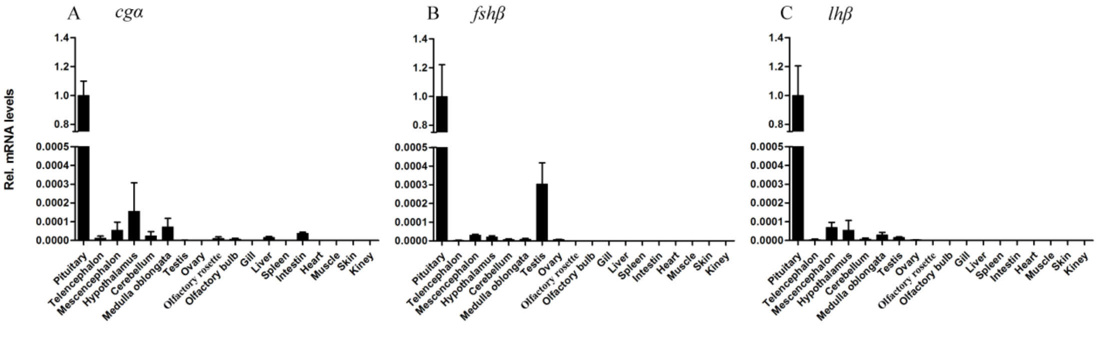

Supplemental Fig.4 Tissue distributions of *cgα* (A), *fshβ* (B) and *lhβ* (C) in *B. sinensis*. The levels of the respective mRNAs were determined using qPCR and normalized to the internal housekeeping gene (*β-actin*). Data are expressed as the mean ± SEM (n = 4).

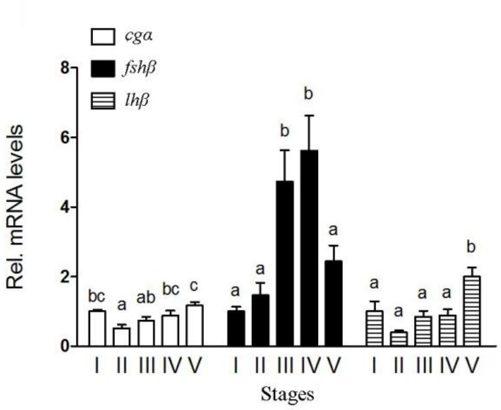

Supplemental Fig.5 Expression of *cgα*, *fshβ* and *lhβ* in the testis during spermatogenesis. Stage I (Spermatogonial proliferation stage), Stage II (Early meiotic stage), Stage III (Mid meiotic stage), Stage IV (Late meiotic stage) and Stage V (Maturation stage). Data are expressed as the mean ± SEM (n=6). Bars marked with different letters are significantly different from each other ($p < 0.05$).

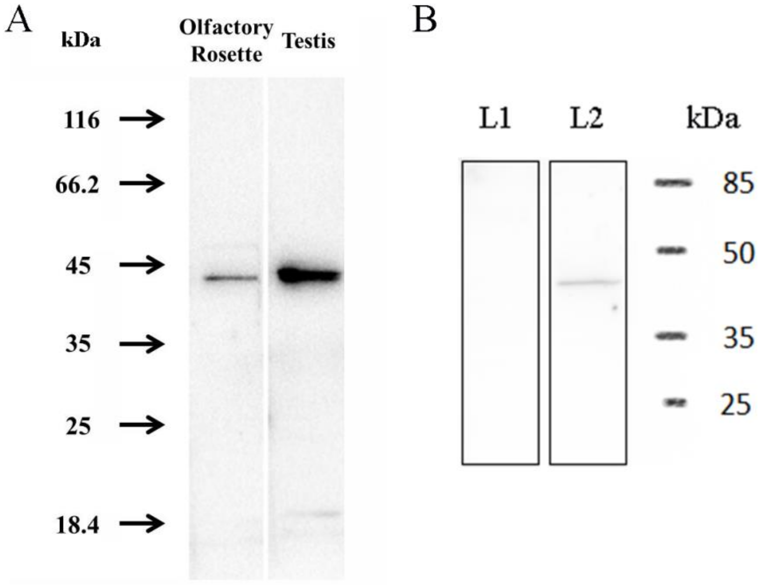

Supplemental Fig.6 Specificity analysis of *B.sinensis* Paqr8 antibody. (A) Western blot of the Paqr8 of *B.sinensis* olfactory rosette and testes. Sample types were indicated on the top of the figure. An unique band at ~41 kDa was seen. (B) Western blot analysis of HEK293T cell transfected with pcDNA plasmid (L1) or Paqr8-pcDNA plasmid (L2). An expected band at ~41 kDa was seen in L2.

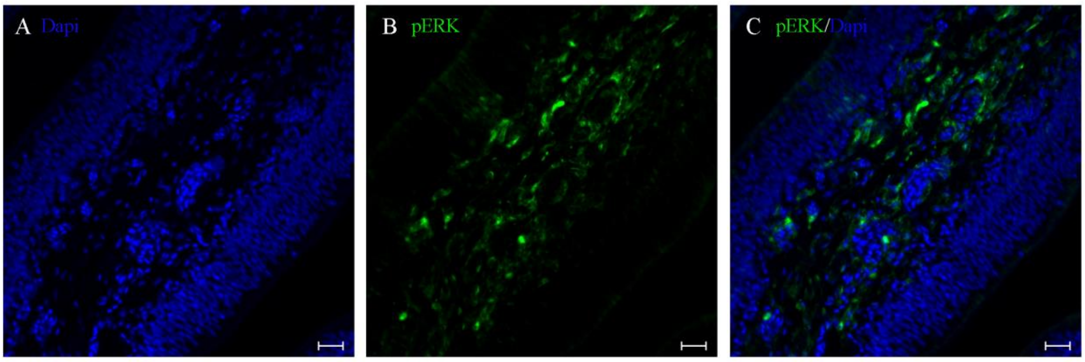

Supplemental Fig.7 pERK immunostaining of the olfactory rosette sections exposed to ethanol. Few positive signals was observed in olfactory sensory neurons. Scale bar=20μm
