# Supplementary material for: Involvement of Membrane Progestin Receptor Beta (mPRβ/Paqr8) in Sex Pheromone Progestin-Induced Expression of Luteinizing Hormone in the Pituitary of Male Chinese Black Sleeper (Bostrychus Sinensis)
Source: Front Endocrinol (Lausanne). 2018 Jul 18;9:397. doi: 10.3389/fendo.2018.00397 (PMC6058016; doi:10.3389/fendo.2018.00397)
Supplement: Supplementary file 2 [file Table_1.docx]

Supplemental table 1, PCR primers used for the cloning and gene expression analyses.

| Genes name | Primer name | Primer sequence (5’-3’) |
| --- | --- | --- |
| Cloning |  |  |
| *cgα* | Forward | TTCTGTAAAATCCAGAGGGATGTCAC |
|  | Reverse | CAATTAGGATACAAGAGGGCTTCCAT |
|  | 3’GSP | TACATTGTGGACACGTACTCCAACAG |
|  | 5’GSP | CAATTAGGATACAAGAGGGCTTCCAT |
|  | 5’GSNP | CCCGAATGCCACCCAACTCTCATAGCT |
| *fshβ* | Forward | CATGTTCTCAGGCCGTTCAG |
|  | Reverse | AACCACAAGCACCAAGTCAA |
|  | 3’GSP | GTCGTGGCGACAGTGTTGGCGTT |
|  | 5’GSP | CAGCCGACGTAGCTGTGGTACACC |
| *lhβ* | Forward | TCCACTGGAGACGCACAG |
|  | Reverse | TTGACTTGGTGCTTGTGGTT |
|  | 3’GSP | CAGTGCACATAGGAAGCATGTCCCCATT |
|  | 5’GSP | GGATGTCGTTCATGCAGAAGTTTGGC |
| qPCR |  |  |
| *β-actin* | Forward | GACAGGTCATCATCATTGGC |
|  | Reverse | CAGACAGCACAGTGTTGGCATAC |
| *cgα* | Forward | GTCCAGTCTATCAATGTATGGGC |
|  | Reverse | TGCCACCCAACTCTATCTCA |
| *fshβ* | Forward | GTGTCGCCCGATGAACATG |
|  | Reverse | GTCTCCACCACACACCTTCT |
| *lhβ* | Forward | TCTCTCCATCTTGTCCGTGC |
|  | Reverse | ACGTCCGGTAGAAGAAGTCC |
| *paqr5* | Forward | TTTTGACTACATCGGCCACA |
|  | Reverse | TTTGTCACCAAGCAAAGCAG |
| *paqr6* | Forward | ATCTGCGTGTACCCCTTCAC |
|  | Reverse | CAGAGCCACAGGGACAAAGT |
| *paqr7a* | Forward | CACATTTCTTCGGGCTATCG |
|  | Reverse | GCTGTCGTTCACAAAGTCCA |
| *paqr7b* | Forward | TTCCCTGTACCGTCAAGCTT |
|  | Reverse | GGGCTGGGCGTGAGGATCCCG |
| *paqr8* | Forward | AGCCAAGTCCAGATATCGCA |
|  | Reverse | GAAGAAGAGGGCTGACGAGA |
| *paqr9* | Forward | ACAATCAGCGCCTATACCGT |
|  | Reverse | CATTTCTGTCGGGTGTTGCA |
| *pgr* | Forward | ATGTGCCTCACATCCTGCTC |
|  | Reverse | GCTGCCTGACTCTTCAGTCC |
| *pgrmc1* | Forward | GCTGTGAACGGGAAAGTGTT |
|  | Reverse | ACTTAAAGGTGAACTGCTGCTC |
| *pgrmc2* | Forward | AAATCTTCGACGTGACCAGC |
|  | Reverse | TGAACTGCATCTCCCACTCC |
